# Microarray-based gene expression profiles in multiple tissues of the domesticated silkworm, *Bombyx mori*

**DOI:** 10.1186/gb-2007-8-8-r162

**Published:** 2007-08-04

**Authors:** Qingyou Xia, Daojun Cheng, Jun Duan, Genhong Wang, Tingcai Cheng, Xingfu Zha, Chun Liu, Ping Zhao, Fangyin Dai, Ze Zhang, Ningjia He, Liang Zhang, Zhonghuai Xiang

**Affiliations:** 1 The Key Sericultural Laboratory of Agricultural Ministry; the Key Laboratory for Sericultural Sciences and Genomics of the Ministry of Education, College of Biotechnology, Southwest University, Beibei, Chongqing 400715, China; 2 National Engineering Center for Beijing Biochip Technology, Life Science Parkway, Changping District, Beijing 102206, China

## Abstract

We designed and constructed a genome-wide microarray with 22,987 70-mer oligonucleotides covering the presently known and predicted genes in the silkworm genome, and surveyed the gene expression in multiple silkworm tissues on day 3 of the fifth instar. Clusters of tissue-prevalent and tissue-specific genes and genes that are differentially expressed in different tissues were identified, and they reflect well major tissue-specific functions on the molecular level. The data presented in this study provide a new resource for annotating the silkworm genome.

## Abstract 2

Using a genome-wide oligonucleotide microarray, gene expression was surveyed in multiple silkworm tissues on day 3 of the fifth instar, providing a new resource for annotating the silkworm genome.

## Background

The silkworm, *Bombyx mori*, is one of the most important insects economically, being employed in the production of silk. The production value of China's silk industry in 2006 reached 17.7 billion dollars [1]. Because of its large size, complex metabolism and the abundance of mutants, it is also one of the best-characterized models for biochemical, molecular genetic and genomic studies of the order Lepidoptera [2,3]. Also, transgenic silkworms can be used as bioreactors for the production of proteinaceous drugs and specific biomaterials. Recently, over 150,000 expressed sequence tags (ESTs) for silkworm have been sequenced, and the draft genome sequences, derived from the silkworm strains *Dazao *and *p50*, respectively, have been finished [4,5]. To date, The BGI Gene Finder program predicted that the silkworm genome encodes roughly 18,510 genes [4]. However, although about 90% of all the available ESTs were contained in the silkworm draft sequence, only 15% of the predicted genes were confirmed by corresponding ESTs. Thus, a genome-wide analysis of gene expression in silkworm will not only estimate the number of transcribed genes in a surveyed tissue, but will also provide evidence for expression of genes without experimental support. Importantly, this approach may provide clues for elucidating the functions of genes underlying specific processes and identifying candidate genes predicted to regulate traits of interest.

DNA microarray technology is an excellent and high-throughput method for measuring the gene expression levels of thousands of genes at the whole-genome scale. This approach has been applied extensively to establish gene expression patterns of many different organisms, including yeast, worm, human, rat, fruit fly and rice [6-11]. Previously, investigators have analyzed gene expression in silkworm wing discs at different time points during metamorphosis using cDNA microarrays constructed from approximately 5,000 ESTs [12,13], and in eggs at different stages during early embryonic development using a cDNA microarray containing 2,445 unique ESTs [14]. Now, the availability of the silkworm whole genome sequence provides the information necessary to design a whole-genome microarray for characterizing genome-wide gene expression in the silkworm.

Here, for the first time, we describe the design of a whole-genome oligonucleotide microarray covering the presently known and predicted genes in silkworm and its application in monitoring gene expression profiles in multiple tissues on day 3 of the fifth instar. This work was intended to survey variation in gene expression across multiple normal silkworm tissue types, to provide experimental evidence for gene function assignments, and to identify gene clusters related to economic traits of interest and to specific cellular processes. Furthermore, the comparison of transcriptomes between silkworm and *Drosophila *provided new insights into the similarities and differences of gene expression in corresponding tissues from the two species.

## Results and discussion

### The validation of microarray data for the transcriptome in multiple silkworm tissues

We designed and constructed a microarray using 22,987 oligonucleotide 70-mer probes representing the presently known and predicted genes in the silkworm genome, and intended to apply it to investigate the silkworm transcriptomes at the whole-genome level. In the present study, we analyzed transcriptional levels in ten representative silkworm tissues (these tested samples mostly belong to tissue/organ type, with the exception of the head and integument; for convenience of description, we consider every selected sample as a tissue; see Materials and methods) from silkworm individuals on day 3 of the fifth instar, using our genome-wide microarray. A typical example of microarray hybridization and its dye-reversal images are viewable in Additional data file 1. In all, we carried out 30 two-channel hybridizations on slides using paired cDNA samples for selected tissues from males and females. We extracted the single channel intensity instead of the ratio value from each two-channel hybridization for further analysis, a strategy that has been reported as being more flexible and valid previously [15-17]. Using intensity-based analysis, the hybridization signal for each RNA sample can be fully utilized in the further statistical analysis, whereas ratio-based analysis with one common reference RNA will double the number of arrays for the identical number of RNA samples.

We evaluated the quality of our microarray data and its consistency with current knowledge of silkworm physiology and gene expression in three different ways. First, we detected the expected expression patterns for genes that were confirmed previously to be expressed specifically in a surveyed tissue type. Genes previously reported as tissue-specific give us an opportunity to verify their tissue-specific expression in our microarray data. We selected those genes whose expression patterns have been profiled in at least the same tissues used in our study. Finally, only four known genes (*ser1*, *BmAHA*, and those encoding beta-glucosidase and tektin) were suitable for this (Additional data file 2). These data can validate the accuracy of our tissue dissections and indicate that there was little cross-contamination during the experiment. Second, we compared the consistency between microarray measurements and EST estimates in the same tissue. For this, we considered 1,642 tissue-specific genes identified from the microarray data (see description in 'Tissue-specific gene expression' below) as queries to perform a local BLASTn search against the silkworm EST collection from GenBank. The EST sequences originated mainly from tissues of the silk gland, fat body, hemocyte, testis, ovary, integument, malpighian tubule, and midgut. In our study, the corresponding ESTs for a tissue-specific gene were determined based on the following stringent post-processing to ensure quality: BLAST alignments with E-value < 1E-30, nucleotide identity > 85% and length coverage > 70%. Finally, of 349 tissue-specific genes confirmed by silkworm ESTs, 295 (84.5%) were further confirmed by tissue-specific ESTs (including ESTs enriched or only expressed in a tissue). This result shows that the ESTs representing most of the tissue-specific genes identified from microarray data were also specifically detected in the corresponding tissues (Additional data file 3). Third, we checked whether our microarray data were consistent with RT-PCR analysis. We tested for the expected tissue-specific expression of 40 selected genes in surveyed tissues using a single primer pair for each gene. Among the 33 pairs (Additional data file 4) that yielded correct product size, 24 (73%) produced the most exclusive signal intensities in the expected tissues (Additional data file 5). The pattern of end-point products of RT-PCR running on agarose gels showed an intuitional correspondence with that of microarray measurements.

### Definition of the expressed genes

Based on the hybridization results of 12 negative controls from exogenous DNA spotted on the array, and the global signal-to-noise ratio of the arrays, we considered a gene to be expressed in a tissue if its signal intensity exceeded a value of 400 signal intensity units after subtracting the background. This criterion is much more stringent than the typical filter threshold determinations that specify the local intensity value should exceed the local background by two standard deviations. As a result, a total of 10,393 active transcripts, which also define active genes, satisfied this criterion in at least one tissue. The number of active genes in each tissue is listed in Table 1. To our knowledge, our work herein represents the largest data set for the analysis of global gene expression in silkworm profiled in a single study.

**Table 1:** Summary of transcribed genes for surveyed tissue types

Tissue	Active genes	Tissue-specific genes (% active genes)
Hemocyte	4,052	24 (0.59%)
Fat body	4,240	6 (0.14%)
A/MSG	4,432	40 (0.90%)
PSG	4,269	13 (0.30%)
Head	6,635	78 (1.18%)
Integument	5,899	14 (0.24%)
Malpighian tubule	5,073	110 (2.17%)
Midgut	5,588	216 (3.87%)
Ovary	6,066	37 (0.61%)
Testis	7,926	1,104 (13.93%)

Our results showed that the microarray analysis is useful for annotating silkworm genes. Of 2,693 transcripts that were detected reliably in all investigated tissues, 306 consistently exceeded 4,000 units and were considered to belong to a house-keeping gene family (for example, ribosomal proteins, actins and tubulins). The remaining 7,700 transcripts were expressed differentially in all selected tissues. In addition, 15% of 21,375 predicted genes were confirmed by silkworm ESTs using a BLASTn search, whereas 44.5% were found to be expressed in selected tissues based on our microarray data.

### Prevalent gene expression patterns

**Figure 1:**
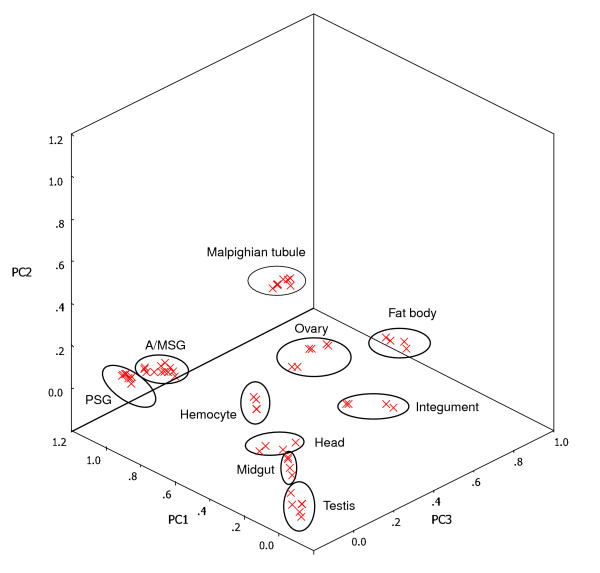
PCA of the silkworm microarray data. The PCA plot illustrates the principal components of all 30 samples from the surveyed tissues. Different individual samples from the same tissue type grouped together, and most tissue types were clearly separated from each other.

After validating the microarray data, we applied principal component analysis (PCA) to assess the internal consistency of different transcriptional data sets from the same tissue. PCA is a mathematical technique used to summarize and visualize the features of the variance in complete microarray data prior to clustering of a set of arbitrarily selected genes [18] or prior to classification of the set [19]. The PCA of all 10,393 active transcripts in surveyed tissues showed that the different data sets from the same tissue types grouped together, and with the exception of the midgut and head, the other surveyed tissues were clearly separated from each other (Figure 1). These results, grouped without prior information, illustrate a strong homogeneity in the pattern of gene expression for each tissue type.

**Figure 2:**
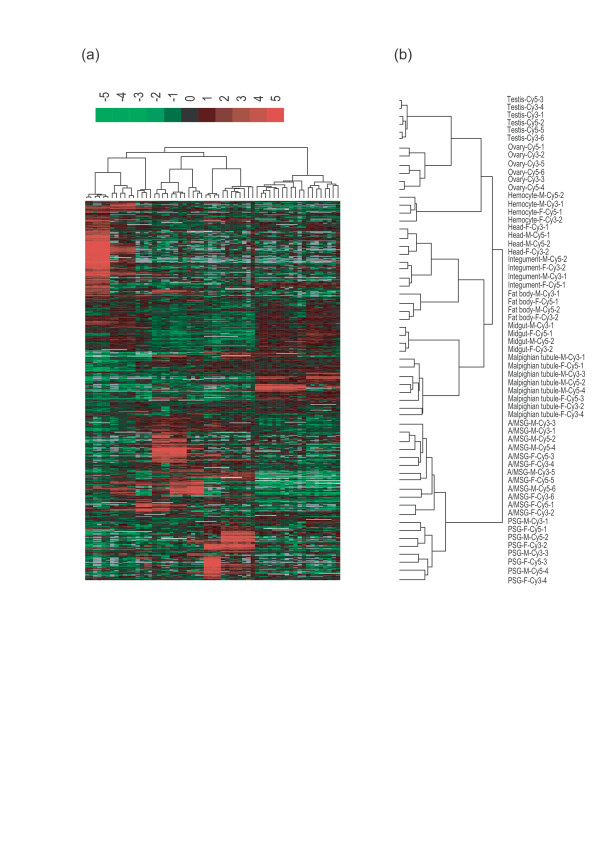
Hierarchical cluster analysis of the surveyed silkworm tissues. **(a) **Overview of the two-way hierarchical cluster of the surveyed tissue types (columns) and 7,700 variably expressed genes (rows). The dataset represented here is available in the BmMDB. **(b) **Enlarged view of the sample dendrogram.

It is common practice to investigate the similarities and differences of gene expression among multiple tissues according to transcription levels by hierarchical clustering (HC). We performed HC and Treeview displays of 7,700 transcripts whose expression varied across the selected tissues, employing the Pearson metric on the averaged expression level values for each tissue. The HC results reflected well that tissues having similar cellular compositions and physiological functions clustered together (Figure 2). For example, the differently functioning parts of the silk gland (anterior/median silk gland (A/MSG) and posterior silk gland (PSG)), the reproductive tissues (testis and ovary), the cuticle-covered samples (head and integument), and the metabolic tissues (midgut and malpighian tubule) grouped together.

**Figure 3:**
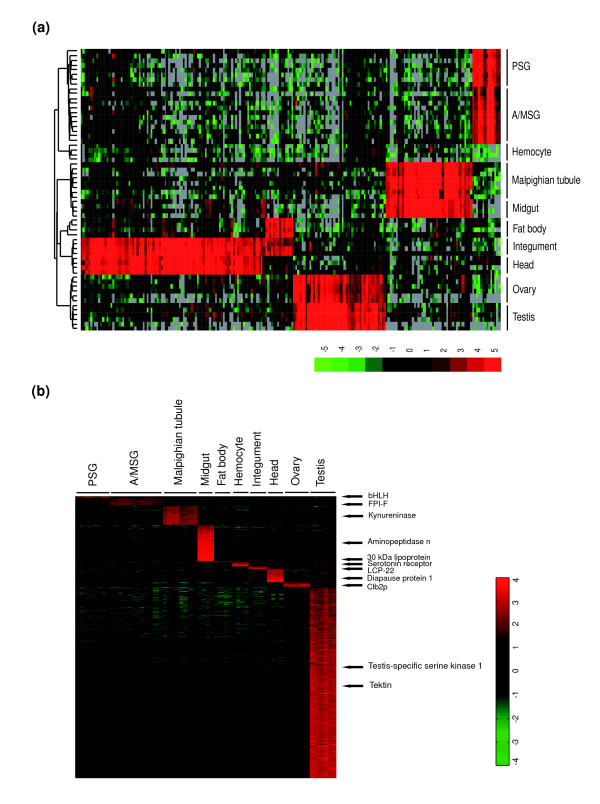
Hierarchical cluster analysis of genes characteristically expressed in different tissue types. **(a) **HC of the selected transcripts that were shared between at least two tissues. **(b) **Heat map of the expression level of all 1,642 tissue-specific genes. These genes were identified by performing pairwise *t*-tests between each tissue and the remaining tissues. Eleven tissue-specific genes are illustrated.

Taken together with Gene Ontology (GO) annotations, the HC analysis also provided clues about how many genes are involved in a biological process shared by at least two surveyed tissue types. Figure 3a shows a heat map of the selected genes that were prevalently expressed in at least two tissues. For example, the head and integument are both surrounded by cuticle and are mainly responsible for signal transduction between outside and inside. Thus, 20 members belonging to the cuticle protein families, which are associated with formation of cuticle, were detected in both samples; genes encoding olfactory proteins and antennal binding proteins are involved in the transduction of the olfactory signals to the central nervous system. In addition, the malpighian tubule and midgut were both characterized by moderate expression of genes associated with metabolism, including those encoding the solute carrier families, aminopeptidase N, carboxypeptidase B precursor, putative inorganic phosphate co-transporter, beta-glucosidase, and low molecular mass 30 kDa lipoprotein 19G1. The testis and ovary showed the highest expression levels of cell cycle- and reproduction- related genes, including those encoding cell cycle checkpoint kinase 2, stathmin, alkylglycerone-phosphate synthase, and egg-specific protein.

### Tissue-specific gene expression

Generally, the tissue-specific gene expression features have been viewed traditionally as predictors of tissue-specific function. In order to identify tissue-specific genes from the 7,700 differentially expressed transcripts, we required that the *P *value for each pairwise *t*-test was less than 0.01 (see Materials and methods), and the intensity for a gene in one surveyed tissue exceeded twice that in other tissues. Finally, 1,642 genes passed these two criteria and were considered to be tissue-specific; the number of genes specifically expressed in each selected tissue displayed a remarkable variation and ranged from 6 in fat body to 1,104 in testis (Table 1). Figure 3b shows the HC heat map of the tissue-specific genes.

The tissue-specific genes exhibited a strong relevance to the physiological functions of the corresponding tissues. For example, the malpighian tubules serve as the excretory and osmoregulatory organs in insects, in which the urine is produced and transported to the hindgut for the selective absorption of water and ions. As expected, of the proteins encoded by tubule-specific genes, we identified various transporters that are responsible for organic cations, monocarboxylates, glucose, and other sugars. Cytochrome P450 molecules and alcohol dehydrogenase participate in metabolism and detoxification in malpighian tubules. In addition, the head is a complex structure containing the most complicated neural ganglia system, which controls biological behavior, including vision and sensory input. Among 78 head-specific gene products, opsin regulates retinal signal transduction; odorant binding protein, chemosensory protein and odorant-degrading esterase are responsible for sensory signaling, and diapause protein 1 and ecdysteroid regulated protein may be related to hormone regulation. Moreover, some integument-specific genes were found to be closely related to cuticle development (for example, those encoding chitin-binding protein and larval cuticle proteins, and *SgAbd-2*, *BMCPA *and *ApCP14*) and to the defense functions of integument (for example, those encoding titin-like protein, scolexin B and proteophosphoglycan, and *Hdd11*). Likewise, we identified two sets of genes that were expressed specifically in fat body and hemocyte. These genes were consistent with metabolic and immune-response functions centered in the fat body, and with the transport of nutrients, hormones and metabolites of the hemocytes, respectively.

### The silk gland transcriptome and its comparison to that of the *Drosophila *salivary gland

The silk gland is the site where silk proteins are synthesized and can be divided into three morphologically and functionally distinct compartments: ASG, MSG and PSG. The PSG exclusively synthesizes the silk fibroin proteins, including fibroin heavy chain, fibroin light chain and fibrohexamerin P25, whereas the MSG yields the glue protein sericins. The ASG is very small compared to the MSG and PSG, and serves as a duct to transport the silk proteins for spinning the cocoons. Because our analysis mainly focuses on the molecular mechanisms for synthesizing and secreting silk proteins, and owing to these properties of the structure and function of the various silk gland regions, we excised the joined ASG and MSG regions together (A/MSG) instead of dissecting the regions separately, and investigated their gene expression patterns in comparison with those in the PSG.

**Figure 4:**
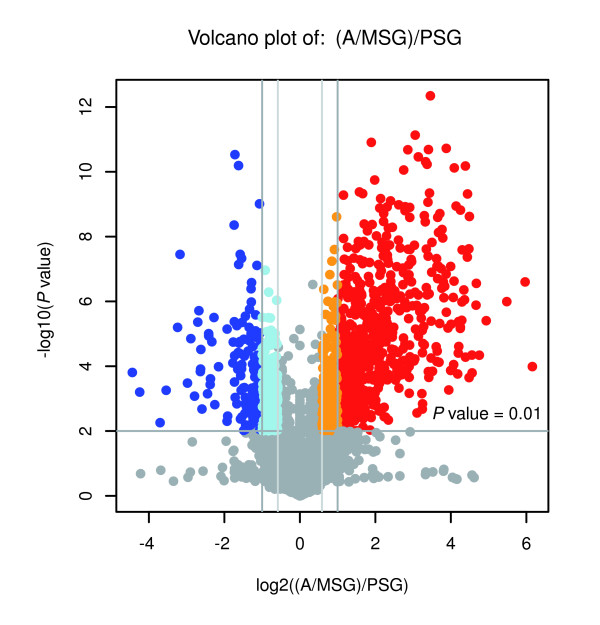
The differential gene expression between A/MSG and PSG. The volcano plot shows the magnitude of differential expression between A/MSG and PSG. Each dot represents one gene that had detectable expression in both tissues. The horizontal line marks the threshold (*P *< 0.01) for defining a gene as up-regulated in PSG (blue) or A/MSG (red), with a combined change > 2-fold. The vertical dashed lines represent two fold differences in expression.

We found that 412 and 109 genes were up-regulated in the A/MSG and PSG, respectively (Figure 4); the GO functional categories for these genes exhibited significant differences (Additional data file 6). For example, aside from the known *ser1 *gene [20], the up-regulated genes in the A/MSG included mainly those encoding proteases, protease inhibitors, dehydrogenases, protein kinases, solute transporters, cuticle proteins and some proteins with unknown function. Of these, protease inhibitors may play an important role in protecting the fibroin proteins in the silk gland lumen against digestion by proteases, such as antennal esterase and serine protease, which are expressed in the A/MSG. On the other hand, a majority of the up-regulated genes in the PSG, besides those encoding the fibroin light chain and fibrohexamerin P25, included mainly those encoding transcription factors, structural proteins, glucose and other sugar transporters, and proteins that aid in hormone signal transduction. For example, a gene encoding the juvenile hormone binding protein precursor-like protein may be involved in juvenile hormone signal transduction in the PSG. Also notable was the up-regulation of a transcription factor containing a basic helix-loop-helix (bHLH) domain in the PSG and expressed specifically in the silk gland. bHLH proteins are known to play a central role in cell proliferation, determination and differentiation [21]. The finding of this silkworm bHLH transcription factor suggests that it might also be responsible for mediating the growth and development of the silk gland.

In addition, we compared the expression patterns between the silk gland of silkworm and the salivary gland of *Drosophila*. Both are typical specialized organs with similar secretory functions, and cellular evidence suggests that they are likely to be homologous [22-25]. In the present study, we found that 34 (13%) of the 255 enriched genes in the larval salivary gland [26] have significant orthologs in the silk gland transcriptome (Additional data file 7). Interestingly, aside from the aforementioned bHLH protein, most of these orthologs are related to gland secretory function and can be classified into two categories. One category mainly consists of secretory pathway component genes, including *srp19*, *sec1*, *sec13*, *sec20*, *sec34*, *spc12*, *and spc25*, which have been demonstrated to have effects on glandular secretion in *Drosophila *[27]. Another category includes genes encoding transporters, such as members of the solute carrier family (family 35 member B3, member E1, and family 39 member 9) and the transmembrane trafficking protein isoform 2, which may be involved in transporting substances relative to silk formation. However, about 87% of the salivary gland-enriched genes did not have corresponding orthologs expressed in the silk gland. Although the cellular evidence implies they are likely to be homologous, our results show that gene expression between the two glands is dramatically different.

### Midgut-specific genes associated with digestion and defense

**Figure 5:**
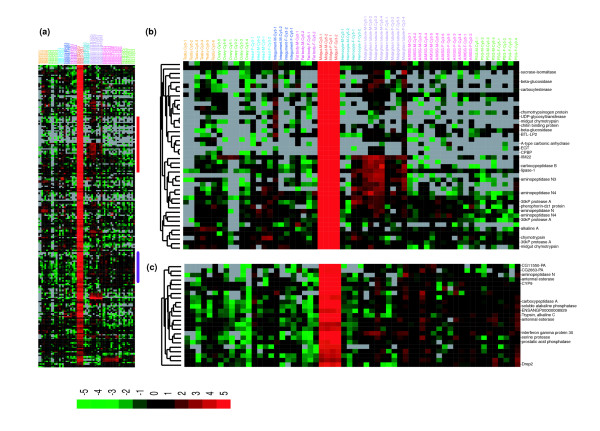
Midgut-specific gene expression. **(a) **Thumbnail overview of a hierarchical cluster of the surveyed tissues and 216 genes specifically expressed in silkworm midgut. Genes are hierarchically clustered while samples are grouped by tissue type. **(b, c) **Hierarchical cluster of selected gene sets with specific molecular functions.

The insect midgut is usually considered to be a location for digestive enzyme synthesis and secretion as well as a major site for nutrient digestion and absorption. Our results show that 216 of 5,588 genes expressed in the midgut displayed tissue-specificity (Figure 5; Additional data file 8). Most of the midgut-specific genes encode enzymes, hydrolase, binding proteins, transferases and transporters, which are mainly involved in the digestion of mulberry leaves, the sole food source for the silkworm, as well as the absorption of nutrients. The gene sets encoding alpha and beta glucosidases, glycoside hydrolase, and glucose transporters are all involved in glucose hydrolysis and transport [28]. Serine proteases, such as endopeptidases and carboxypeptidases A and/or B, hydrolyze nutrient proteins into amino acids that can be absorbed and utilized by insect gut cells [29]. Additionally, the genes encoding the lipase protein family, antennal esterases, carboxylesterase, and scavenger receptor SR-B1 were associated mainly with lipid metabolism, such as the hydrolysis of triglycerides, degradation of odorant acetate compounds, and the binding of modified low-density lipoproteins.

The midgut also represents the first line of resistance and immune response of the silkworm. Genes encoding aminopeptidases were enriched in the midgut; aminopeptidases function as Cry toxin receptors to bind differentially the various classes of the Cry toxins. BtR175, a cadherin-like protein expressed in the silkworm, triggers Cry1Aa-induced cell lysis in a baculovirus gene expression system, suggesting that it may also function as a Cry toxin receptor [30]. Also expressed in the midgut were 17 members of the cytochrome P450 gene family, including *CYP4*, *CYP6 *and *CYP9*, which may be involved in metabolism of plant toxins and insecticides; of these, only *CYP6x *and *CYP9 *were expressed specifically in the midgut (Figure 5c). Another midgut-specific gene encodes peptidoglycan recognition protein, which can bind strongly to the cell wall peptidoglycans of Gram-positive bacteria and trigger the immune response. Furthermore, we found that two lymphocyte receptor genes were expressed specifically in the midgut, suggesting that they may encode binding proteins that function in the recognition of pathogens [31].

**Figure 6:**
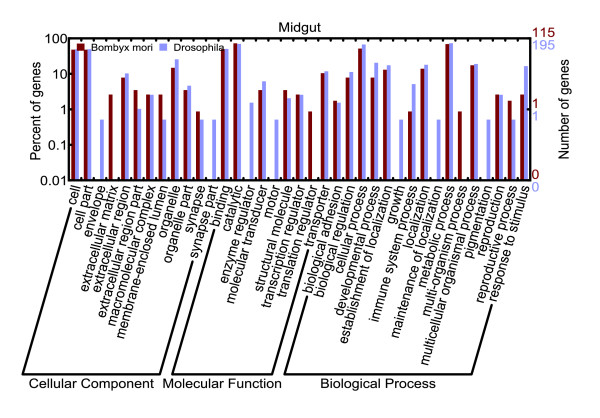
GO categories of midgut-specific genes from late larval silkworm and *Drosophila*. Although few orthologous genes were identified in the midgut-specific transcriptome between silkworm and *Drosophila*, the differences between the two sets of midgut-specific genes were small on the basis of GO annotation.

Additionally, orthologs corresponding to 30 of 313 midgut-enriched genes in *Drosophila *[26] were identified in the silkworm midgut transcriptome. Of these, only one, encoding soluble alkaline phosphatase, was expressed uniquely in the silkworm midgut, whereas the remaining orthologs were also detected in other tissues. This indicated that the silkworm and fruit fly have two different sets of midgut-specific genes. The difference may be due to the different feeding and living habits of these two insects, since silkworms eat only mulberry leaves and flies subsist on a wide variety of foods. However, the GO functional annotations for the two sets were similar (Figure 6), suggesting that these genes may have similar biological functions or be involved in similar physiological processes.

### Sexual dimorphism of gene expression in the silkworm

Sexual dimorphism is the difference between sexes of the same species. This dimorphism is initiated by the components of the sex determination pathway and results in sex-dimorphic differences of gene expression in various tissues, such as gonads (testis and ovary) and other somatic tissues [32-34]. Silkworm is a female-heterogametic organism (*ZZ *in male, *ZW *in female). The homologs for more than ten sex-determining factors from *Drosophila *were identified by searching the silkworm 6× draft sequence [4]. However, the extent of sexual dimorphism of gene expression in a variety of silkworm tissues has not been fully examined. Therefore, a systematic understanding of sexually dimorphic genes and the mechanisms underlying differences between sexes are of great interest. In all, we observed that significantly more genes were up-regulated in male gonad, malpighian tubule and integument. This situation was reversed in the head (Table 2).

**Table 2:** Distribution of genes differentially expressed between sexes

Tissue	Active genes	Dimorphic genes	Up-regulated in female	Up-regulated in male
Gonad	8,542	2,462 (28.8%)	217 (2.5%)	2,245 (26.3%)
Hemocyte	4,052	89 (2.2%)	56 (1.4%)	33 (0.8%)
Fat body	4,240	71 (1.7%)	27 (0.6%)	44 (1.0%)
Head	6,635	105 (1.6%)	82 (1.2%)	23 (0.3%)
PSG	4,269	19 (0.4%)	15 (0.4%)	4 (0.1%)
Malpighian tubule	5,073	231 (4.6%)	68 (1.3%)	163 (3.2%)
Midgut	5,588	47 (0.8%)	25 (0.4%)	22 (0.4%)
A/MSG	4,432	10 (0.2%)	5 (0.1%)	5 (0.1%)
Integument	5,899	255 (4.3%)	14 (0.2%)	241 (4.1%)

The differences in gene expression between testis and ovary were most striking. Differentially expressed genes were enriched for motor activity, binding and catalytic activity, signal transduction, and transport. Of these, 1,104 genes were expressed specifically in the testis, and were associated with spermatogenesis, reproduction, mitosis and fertilization. For example, testis-specific serine kinase 1, tektin, and testis spermatogenesis apoptosis-related protein have been found to be involved in spermatogenesis [35,36]. Moreover, the male silkworm undergoes meiotic recombination whereas the female does not. This situation is reversed in *Drosophila*. In both the microarray survey and independent RT-PCR confirmation we observed testis-specific expression of a homolog of a key gene encoding a meiotic recombination protein, SPO11, which initiates meiotic recombination [37,38]. Nevertheless, the *Drosophil*a homolog of SPO11, *mei-W68*, has been confirmed to be expressed at a higher level in ovary than in testis [39]. These observations suggest that SPO11 is a candidate for playing a key role in meiotic recombination of the male silkworm, and of female *Drosophila*.

In somatic tissues, many sexually dimorphic genes were enriched for catalytic activities, metabolic pathways, immune/defense responses and transport. Of these, aside from a few that have been confirmed in previous studies [40,41], most were the first to be identified as sexually dimorphic on the basis of microarray data. Interestingly, several gene families showed sexually distinct expression patterns in certain tissues. For example, genes encoding two sets of heat shock proteins, hsp70, hsp20.8 and hsp19.9 in A/MSG, and hsp90, hsp70, hsp20.8, hsp20.1, hsp19.9 and hsp70 cognate in the PSG, were found to be up-regulated in the female. These results are in agreement with the expression model of hsp72 in rat [42]. In the midgut, head and integument, the genes encoding low molecular mass 30 kDa lipoproteins that function in defense and as a nutrient reservoir [43] were up-regulated in the male. All of these findings indicate there is somewhat different behavior and physiologies between silkworm sexes, and further analysis of all sexually dimorphic genes will provide new insights into the molecular mechanisms for regulating these differences.

## Conclusion

We have surveyed gene expression across multiple silkworm tissues by means of a whole-genome oligonucleotide microarray. This is the first study to analyze gene expression profiles of normal silkworm tissues on a genomic scale. Based on our microarray data, we identified tissue-prevalent and tissue-specific genes and genes that are differentially expressed in different tissues. The comparative analysis of the transcriptome in corresponding tissues from silkworm and fruit fly revealed some interesting features. Our microarray-based data of gene expression provide a baseline for prioritizing follow-up experiments aimed at elucidating the molecular functions of various tissues and for annotating more completely the silkworm genome. We have constructed a *Bombyx mori *Microarray Database (BmMDB) and web-browser to store the silkworm gene expression data (Additional data file 9) [44]. The BmMDB will be open to the public soon. This data set will also serve as a reference that can be used by other investigators to compare with their own data, and to further characterize genes of interest and design new experiments.

## Materials and methods

### Oligonucleotide microarray design and construction

All sequences that were regarded as models for designing probes were derived from the draft silkworm genome sequence database [45] and other resources available in GenBank. The data set contained 21,375 predicted genes (21,302 reported in the previous study [2] and 73 newly identified genes) from the silkworm whole genome sequence and 1,612 ESTs of interest that were not contained in the predicted genes.

The 70-mer oligonucleotide probes were designed by CapitalBio Corporation (Beijing, China) and were synthesized by MWG Biotech (Ebersberg, Germany). All oligonucleotide probes were modified at the 5' ends with a six-carbon linker and a primary amine corresponding to each sequence. The probes were dissolved in EasyArray™ spotting solution (CapitalBio Corp.) at 40 μM and finally spotted on aminosilane coated slides using a SmartArray™ microarrayer (CapitalBio Corp.). On one slide there were 48 blocks, and every block had 23 columns and 22 rows. The probes for four house-keeping genes and eight yeast intergenic sequences were spotted on every block as internal and external controls, respectively.

### Experimental silkworm tissues and RNA isolation

The Chinese silkworm strain *Dazao *was reared at a stable temperature of 25°C. The silkworm feeds and grows quickly during its entire larval stage. The larvae stopped feeding and began spinning the cocoon on day 7 of the fifth instar. Day 3 of the fifth instar is the boundary for larval development. Most biological processes may be similar during successive feeding stages at and before this time point, but after it silkworms begin to synthesize mass silk proteins in the silk gland, which grows rapidly [46]. Thus, the study of this time point will be helpful to elucidate the regulatory mechanism of the mass synthesis of silk proteins and the growth of silkworm larva as well. In the present study, we surveyed gene expression in the A/MSG, the PSG, testis, ovary, fat body, midgut, integument, hemocyte, malpighian tubule, and head from silkworm individuals on day 3 of the fifth instar (these tested samples mostly belong to tissue/organ types, with the exception of the head and integument; for convenience of description, we consider each selected sample as a tissue).

In order to establish gene expression differences between sexes, we prepared male and female samples of the same tissue. To obtain enough tissue for the total RNA extractions, we adopted a sample pooling strategy; each tissue was collected from 100 silkworms. In addition, we also selectively performed the biological replicates at least twice for five tissues, including testis, ovary, A/MSG, PSG and malpighian tubule, to evaluate biological reproducibility. In all, we prepared 30 two-channel hybridizations across the selected tissues for study. The dissected tissue samples were snap-frozen and held in liquid nitrogen for RNA extraction. Total RNA was isolated from each sample using TRIzol reagent (Invitrogen, Carlsbad, CA, USA) according to the manufacturer's instructions. The total RNA templates were quantified by spectrophotometer and subjected to 1.0% formaldehyde denatured agarose gel electrophoresis. The average yield of RNA in each sample was approximately 0.5 μg/mg.

### RNA amplification, labeling and hybridization

Total RNA (5 μg) was used to prepare the fluorescent dye-labeled cDNA using the linear mRNA amplification procedure described in previous studies [17,47]. The resulting labeled cDNAs were dissolved in 80 μl of hybridization solution containing 3×SSC, 0.2% SDS, 5×Denhardt's solution and 25% formamide, then denatured at 95°C for 3 minutes before hybridization. The mixed hybridization buffer was loaded onto a microarray slide, and then covered with a LifterSlip™ coverslip (Erie Company, Portsmouth, NH, USA). The hybridizations were performed in a hybridization chamber that was placed on a three-phase tiling agitator (BioMixer™; CapitalBio Corp.) to provide continuous mixing of the hybridization buffer, with more uniform hybridization across the entire slide surface and to prevent edge effects. After hybridization, slides were washed with washing solution 1 and 2 (0.2% SDS, 2× SSC and 2× SSC, respectively) at 42°C for 5 minutes.

We used a dual-dye experiment to analyze the expression patterns; one of the dyes (Cy3) was used to label a female sample of a particular tissue type, while the other dye labeled a male sample of the same tissue type. Each experiment was performed as a dye-swap. In our pilot experiments, we also performed several self-to-self hybridizations to evaluate system noise. Additionally, we adopted a DNA-DNA hybridization protocol to replace the RNA-DNA hybridizations in the present study considering that the former may reduce microarray cross-hybridization [48].

### Microarray imaging and data analysis

Arrays were scanned with a confocal LuxScan™ scanner and the images obtained were then analyzed using LuxScan™ 3.0 software (both from CapitalBio Corp.). For the individual channel data extracts, we removed any faint spots, with signal intensities below 400 units after subtracting the background, from both channels (Cy3 and Cy5).

Considering that global gene expression changes may exist across different silkworm tissues, we applied a linear normalization method to normalize individual channel data by using four confirmed house-keeping genes (those encoding proteasome beta subunit, eIF-3 subunit 4, eIF 3A subunit 5, and eIF 4A) instead of the prevalent LOWESS normalization method for dual-channel microarrays. The normalized signal intensity values were further analyzed using one-way analysis of variance (ANOVA), with the significance level set at a *P *value of less than 0.001 across all investigated tissues. The filtered data were further examined to find genes that are differentially expressed in different tissues if the expression level in one tissue exceeded two-fold that in another tissue, or to identify tissue-specific genes using unpaired *t*-tests with the significance level set at *P *< 0.01, together with a change between one tissue and each of all the other tissues of more than two-fold. In addition, genes expressed in all tissues with intensity values that exceeded an average of 4,000 units were regarded as being potential house-keeping genes. HC with the average linkage method was performed using Cluster software [49]. A reciprocal-best-BLAST-hits approach was applied to predict orthologs between silkworm and *Drosophila*.

### Semi-quantitative RT-PCR confirmation

Primer pairs were designed to have melting temperatures matched to each other. The resulting sequences are listed in Additional data file 4. The RT-PCR confirmation results were performed using the OneStep RT-PCR Kit (Qiagen, Hamburg, Germany) and each reaction was prepared in 25 μl containing 10 ng cDNA, 5 units Taq DNA polymerase, and 10 pM each of the forward and reverse primers. After 30 cycles of amplification, the reaction products were separated on 2% agarose gels stained with ethidium bromide.

### Microarray database construction

We constructed BmMDB to store the silkworm gene expression data [44]. The backend of the database uses MySQL [50] to manage the data. Php scripts are used both to query the database and generate the HTML or heat map picture to display the query results. The database contains the raw data for all experiments, and will be open to the public at the BmMDB. The database can be queried by probe ID; the detailed annotation (for example, the name and coding sequence) of the silkworm gene covered by each probe is also provided in this database, and all the coding sequences of silkworm genes can be searched by an attached BLAST.

## Additional data files

The following additional data files are available with the online version of this manuscript. Additional data file 1 is a figure showing the dye-reversal image for a typical microarray hybridization reaction. Additional data file 2 lists the four genes previously confirmed experimentally to be tissue-specific. Additional data file 3 shows the expression consistency between tissue-specific genes identified from microarray data and their corresponding ESTs. Additional data file 4 lists the primer pairs that were tested for selected genes. Additional data file 5 shows the consistency between microarray identification and RT-PCR confirmation for the selected tissue-specific genes. Additional data file 6 shows the comparison of functional categories for genes differentially expressed in the A/MSG and PSG. Additional data file 7 lists the expressed orthologous genes between silkworm silk gland and *Drosophila *salivary gland. Additional data file 8 lists the midgut-specific genes. Additional data file 9 shows the screenshot of the database for the silkworm microarray data.

## Abbreviations

A/MSG = anterior/median silk gland; bHLH = basic helix-loop-helix; BmMDB = *Bombyx mori *Microarray Database; EST = expressed sequence tags; GO = gene ontology; HC = hierarchal clustering; PCA = principal component analysis; PSG = posterior silk gland.

## Authors' contributions

QYX and ZHX conceived and coordinated the study. QYX, TCC, XFZ and LZ were responsible for the design and production of the microarray. DJC, GHW, CL, PZ and FYD performed the tissue samples preparation, RNA isolation, RNA amplification, labeling, and hybridization. DJ, GHW and LZ participated in the acquisition and statistical analysis of microarray data. QYX, DJC, DJ, GHW, TCC, XFZ and CL were responsible for the interpretation of microarray data. GHW confirmed the microarray data by RT-PCR. DJ constructed the microarray database for searching. QYX drafted the manuscript. ZZ, NJH and LZ reviewed the manuscript. All authors read and approved the final manuscript.

## Supplementary Material

Additional data file 1 Dye-reversal image for a typical microarray hybridization reaction. Click here for file

Additional data file 2 The four genes previously confirmed experimentally to be tissue-specific. Click here for file

Additional data file 3 Expression consistency between tissue-specific genes identified from microarray data and their corresponding ESTs. Click here for file

Additional data file 4 Primer pairs that were tested for selected genes. Click here for file

Additional data file 5 Consistency between microarray identification and RT-PCR confirmation for the selected tissue-specific genes. Click here for file

Additional data file 6 Comparison of functional categories for genes differentially expressed in the A/MSG and PSG. Click here for file

Additional data file 7 Expressed orthologous genes between silkworm silk gland and *Drosophila *salivary gland. Click here for file

Additional data file 8 Midgut-specific genes. Click here for file

Additional data file 9 Screenshot of the database for the silkworm microarray data. Click here for file
