# Supplementary material for: Microarray-based gene expression profiles in multiple tissues of the domesticated silkworm, Bombyx mori
Source: Genome Biol. 2007 Aug 4;8(8):R162. doi: 10.1186/gb-2007-8-8-r162 (PMC2374993; doi:10.1186/gb-2007-8-8-r162)
Supplement: Additional data file 1 — Dye-reversal image for a typical microarray hybridization reaction. [file gb-2007-8-8-r162-S1.ppt]

## Slide 1
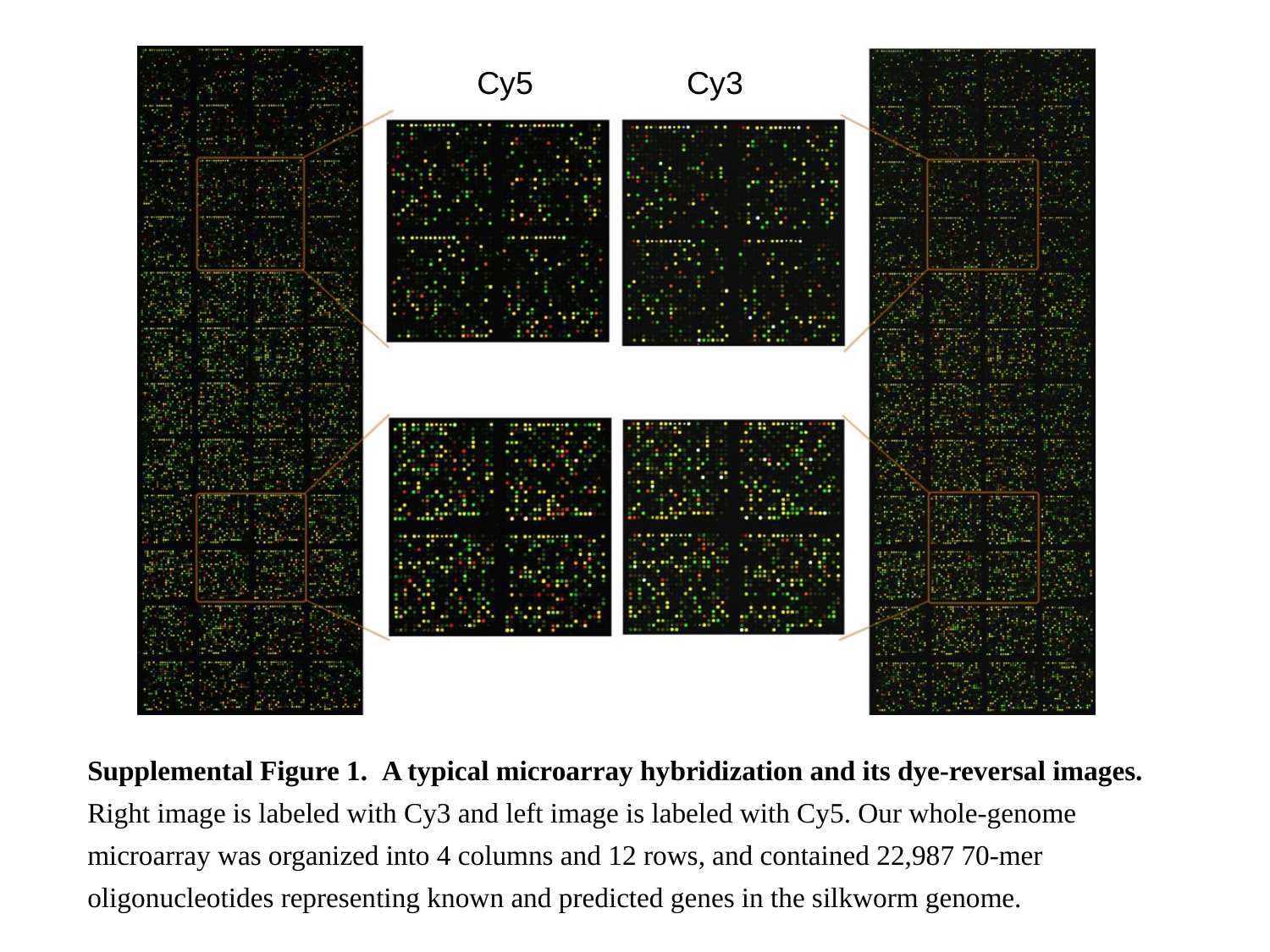

Cy5
Cy3
Supplemental Figure 1. A typical microarray hybridization and its dye-reversal images. Right image is labeled with Cy3 and left image is labeled with Cy5. Our whole-genome microarray was organized into 4 columns and 12 rows, and contained 22,987 70-mer oligonucleotides representing known and predicted genes in the silkworm genome.
