# Supplementary material for: Microarray-based gene expression profiles in multiple tissues of the domesticated silkworm, Bombyx mori
Source: Genome Biol. 2007 Aug 4;8(8):R162. doi: 10.1186/gb-2007-8-8-r162 (PMC2374993; doi:10.1186/gb-2007-8-8-r162)
Supplement: Additional data file 2 — The four genes previously confirmed experimentally to be tissue-specific. [file gb-2007-8-8-r162-S2.ppt]

## Slide 1
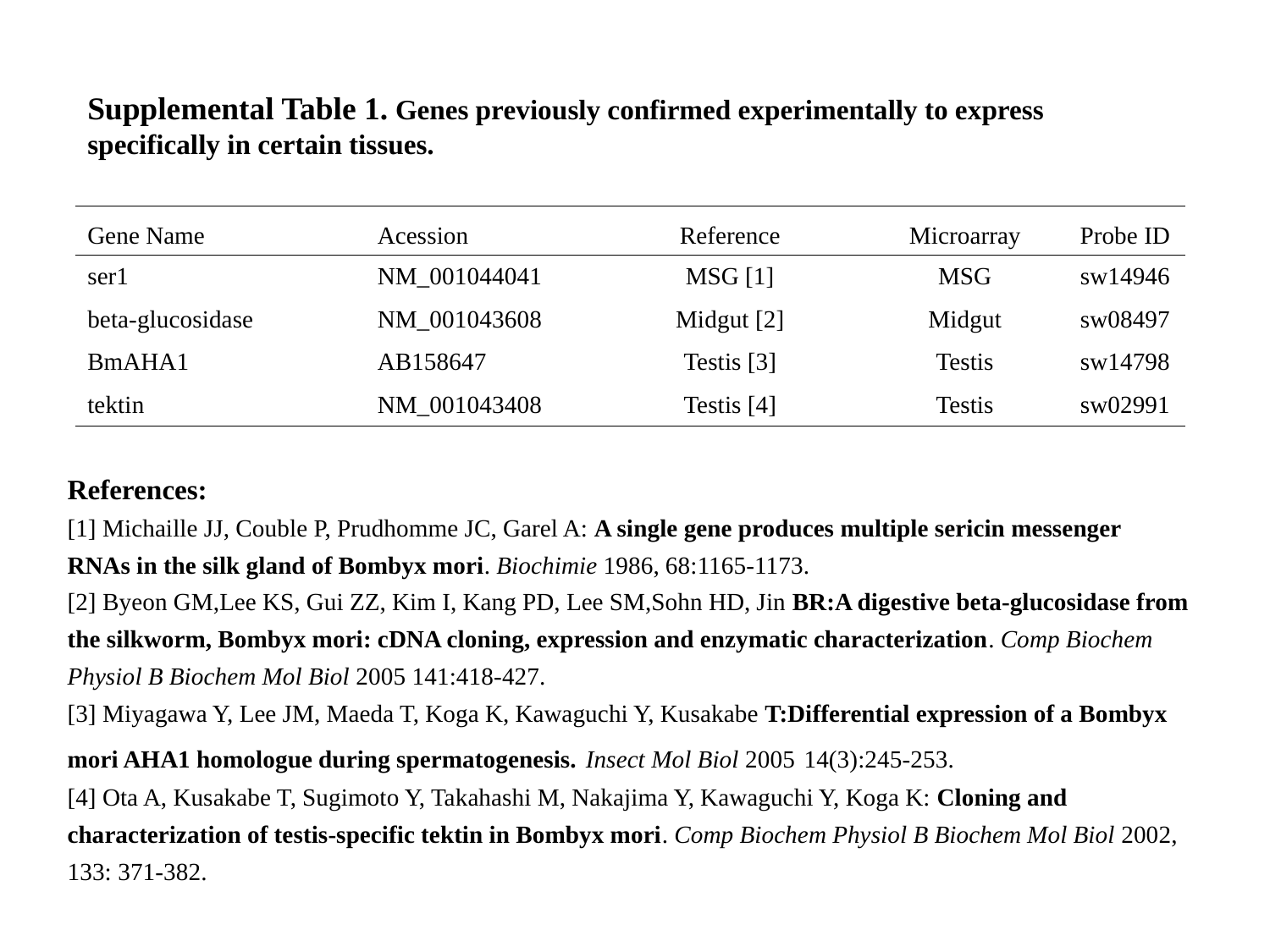

Supplemental Table 1. Genes previously confirmed experimentally to express
specifically in certain tissues.
| Gene Name | Acession | Reference | Microarray | Probe ID |
| --- | --- | --- | --- | --- |
| ser1 | NM\_001044041 | MSG [1] | MSG | sw14946 |
| beta-glucosidase | NM\_001043608 | Midgut [2] | Midgut | sw08497 |
| BmAHA1 | AB158647 | Testis [3] | Testis | sw14798 |
| tektin | NM\_001043408 | Testis [4] | Testis | sw02991 |
References:
[1] Michaille JJ, Couble P, Prudhomme JC, Garel A: A single gene produces multiple sericin messenger RNAs in the silk gland of Bombyx mori. Biochimie 1986, 68:1165-1173.
[2] Byeon GM,Lee KS, Gui ZZ, Kim I, Kang PD, Lee SM,Sohn HD, Jin BR:A digestive beta-glucosidase from the silkworm, Bombyx mori: cDNA cloning, expression and enzymatic characterization. Comp Biochem Physiol B Biochem Mol Biol 2005 141:418-427.
[3] Miyagawa Y, Lee JM, Maeda T, Koga K, Kawaguchi Y, Kusakabe T:Differential expression of a Bombyx mori AHA1 homologue during spermatogenesis. Insect Mol Biol 2005 14(3):245-253.
[4] Ota A, Kusakabe T, Sugimoto Y, Takahashi M, Nakajima Y, Kawaguchi Y, Koga K: Cloning and characterization of testis-specific tektin in Bombyx mori. Comp Biochem Physiol B Biochem Mol Biol 2002, 133: 371-382.
