# Supplementary material for: Microarray-based gene expression profiles in multiple tissues of the domesticated silkworm, Bombyx mori
Source: Genome Biol. 2007 Aug 4;8(8):R162. doi: 10.1186/gb-2007-8-8-r162 (PMC2374993; doi:10.1186/gb-2007-8-8-r162)
Supplement: Additional data file 3 — Expression consistency between tissue-specific genes identified from microarray data and their corresponding ESTs. [file gb-2007-8-8-r162-S3.ppt]

## Slide 1
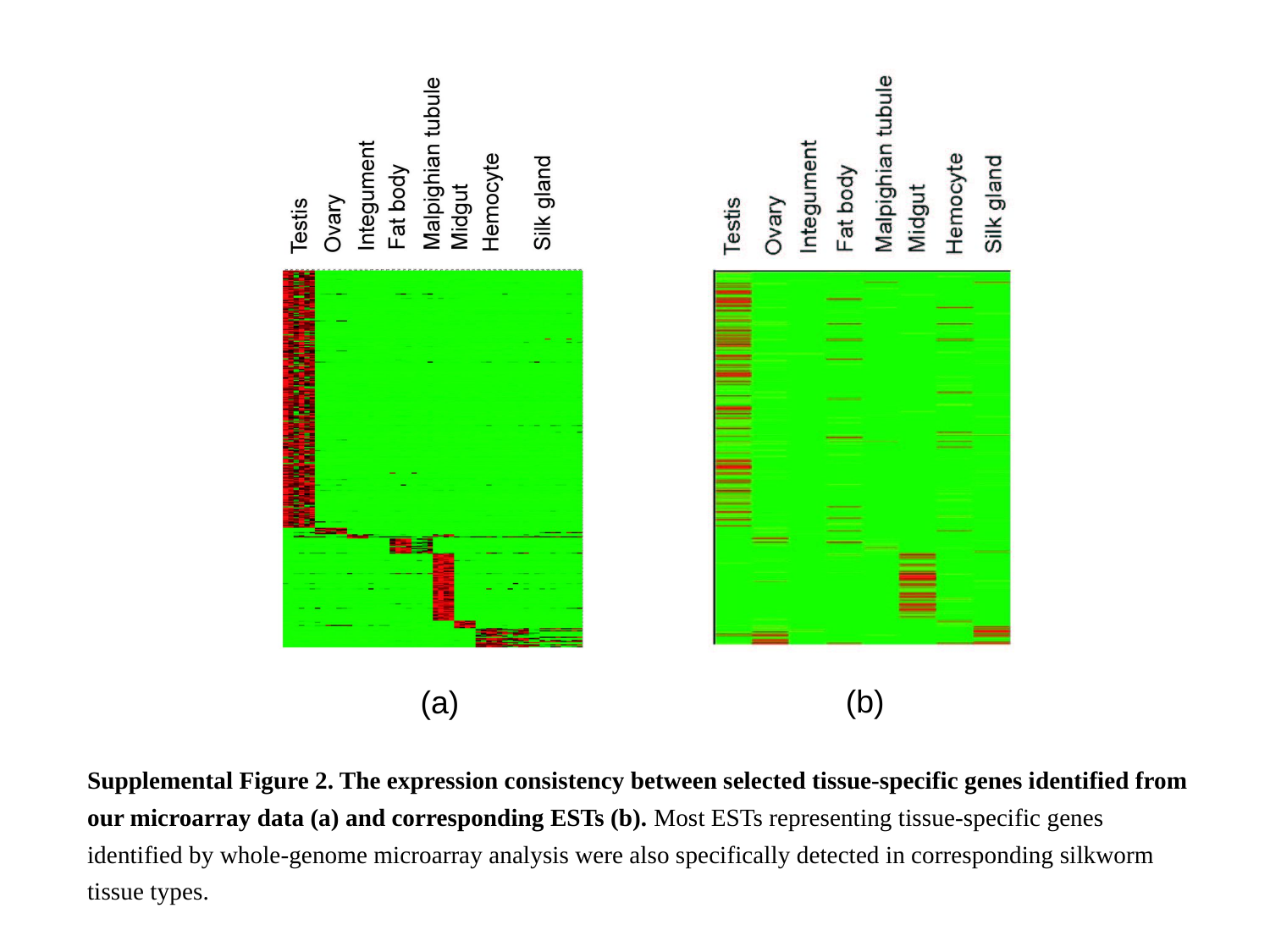

(b)
(a)
Supplemental Figure 2. The expression consistency between selected tissue-specific genes identified from our microarray data (a) and corresponding ESTs (b). Most ESTs representing tissue-specific genes identified by whole-genome microarray analysis were also specifically detected in corresponding silkworm tissue types.
