# Supplementary material for: Microarray-based gene expression profiles in multiple tissues of the domesticated silkworm, Bombyx mori
Source: Genome Biol. 2007 Aug 4;8(8):R162. doi: 10.1186/gb-2007-8-8-r162 (PMC2374993; doi:10.1186/gb-2007-8-8-r162)
Supplement: Additional data file 5 — Consistency between microarray identification and RT-PCR confirmation for the selected tissue-specific genes. [file gb-2007-8-8-r162-S5.ppt]

## Slide 1
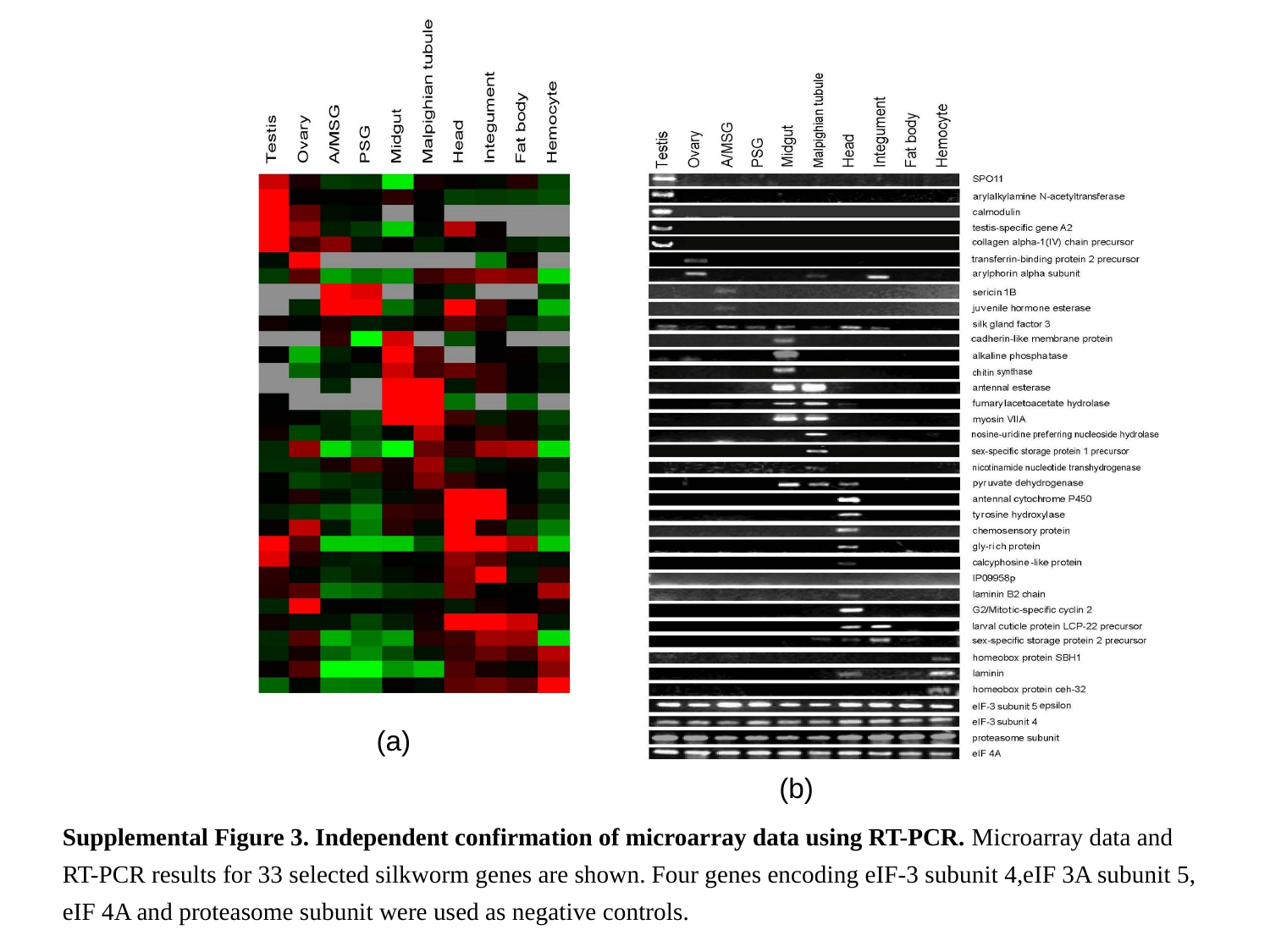

(a)
(b)
Supplemental Figure 3. Independent confirmation of microarray data using RT-PCR. Microarray data and RT-PCR results for 33 selected silkworm genes are shown. Four genes encoding eIF-3 subunit 4,eIF 3A subunit 5, eIF 4A and proteasome subunit were used as negative controls.
