# Supplementary material for: Microarray-based gene expression profiles in multiple tissues of the domesticated silkworm, Bombyx mori
Source: Genome Biol. 2007 Aug 4;8(8):R162. doi: 10.1186/gb-2007-8-8-r162 (PMC2374993; doi:10.1186/gb-2007-8-8-r162)
Supplement: Additional data file 6 — Comparison of functional categories for genes differentially expressed in the A/MSG and PSG. [file gb-2007-8-8-r162-S6.ppt]

## Slide 1
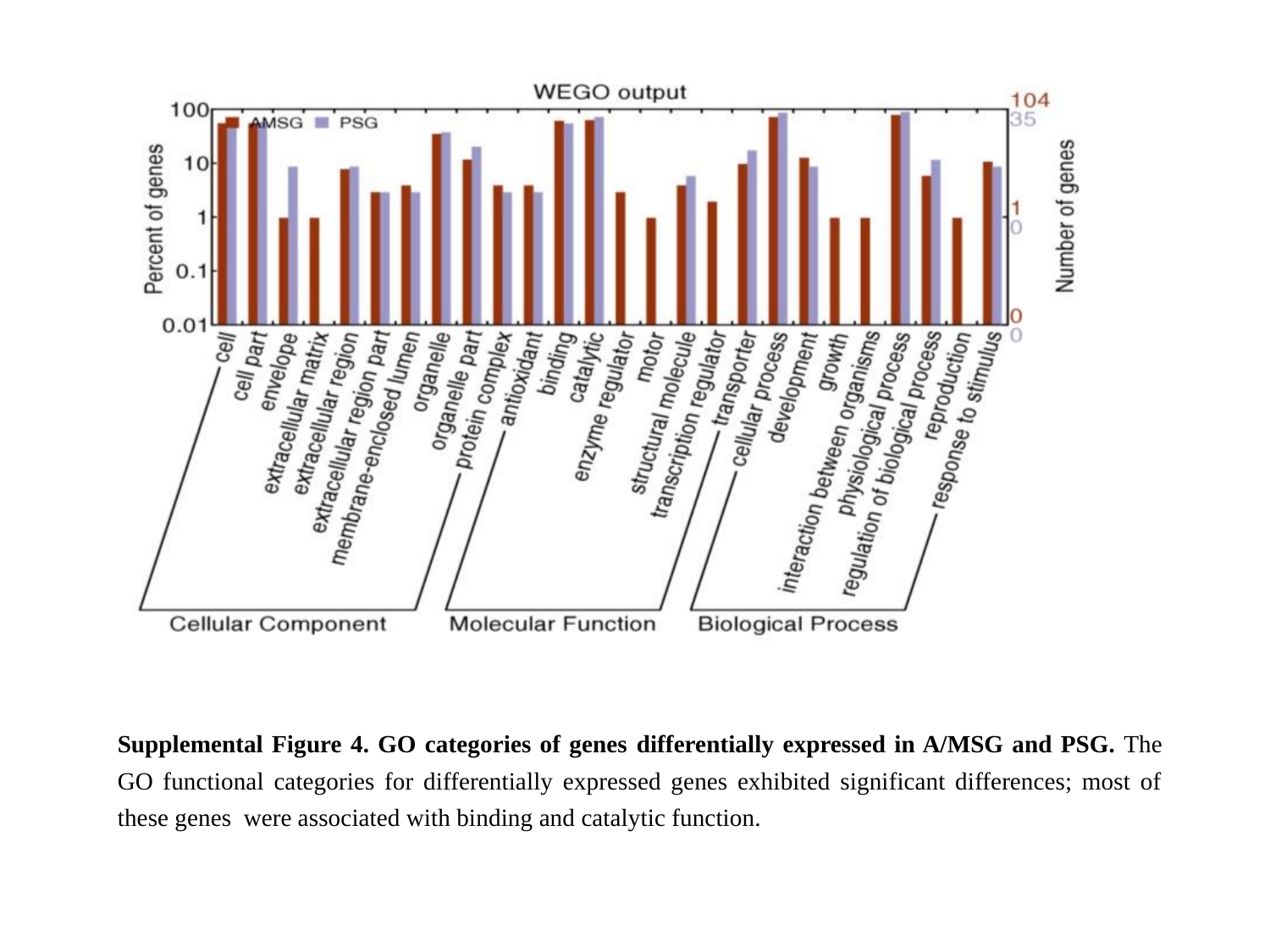

Supplemental Figure 4. GO categories of genes differentially expressed in A/MSG and PSG. The GO functional categories for differentially expressed genes exhibited significant differences; most of these genes were associated with binding and catalytic function.
