# Supplementary material for: Microarray-based gene expression profiles in multiple tissues of the domesticated silkworm, Bombyx mori
Source: Genome Biol. 2007 Aug 4;8(8):R162. doi: 10.1186/gb-2007-8-8-r162 (PMC2374993; doi:10.1186/gb-2007-8-8-r162)
Supplement: Additional data file 9 — Screenshot of the database for the silkworm microarray data. [file gb-2007-8-8-r162-S9.ppt]

## Slide 1
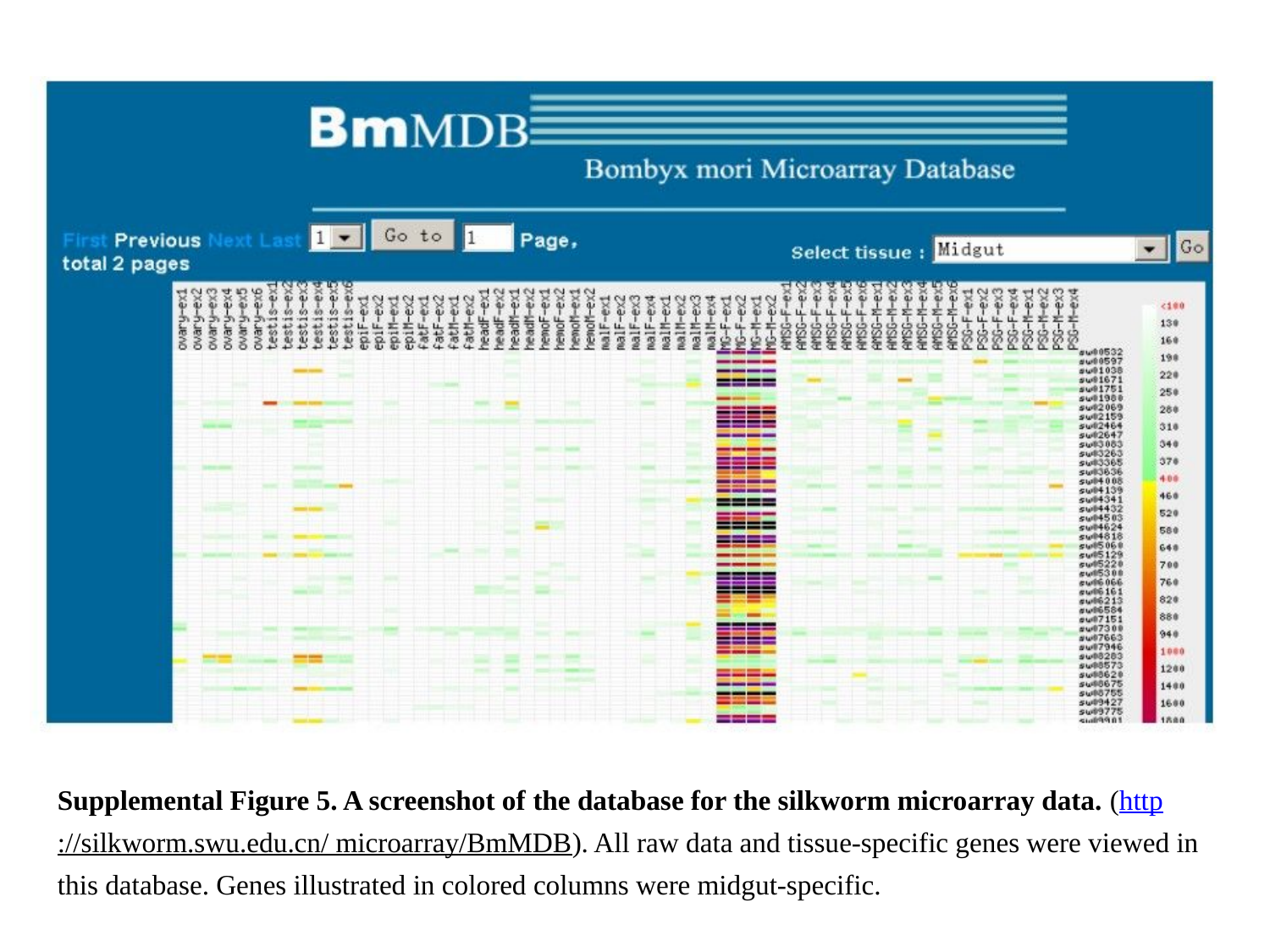

Supplemental Figure 5. A screenshot of the database for the silkworm microarray data. (http://silkworm.swu.edu.cn/ microarray/BmMDB). All raw data and tissue-specific genes were viewed in this database. Genes illustrated in colored columns were midgut-specific.
